# Sodium salicylate reduced mRNA abundance of hypoxia-associated genes in MAC-T cells

**DOI:** 10.3168/jdsc.2020-0037

**Published:** 2021-03-26

**Authors:** C.M. Ylioja, T.H. Swartz, L.K. Mamedova, B.J. Bradford

**Affiliations:** 1 Department of Animal Sciences and Industry, Kansas State University, Manhattan 66506; 2 Department of Animal Science, Michigan State University, East Lansing 48824

## Abstract

Hypoxia is an oxygen deficiency commonly found in growing tissues and is speculated to occur in the rapidly developing mammary gland in peripartum dairy cattle. Low oxygen concentrations can activate hypoxia-inducible factor-1 (HIF-1), which increases transcription of genes involved in angiogenesis (*VEGFA*) and glucose transport (*GLUT1*), among other processes. The mRNA stability of these genes is positively regulated by heterogeneous nuclear ribonucleoprotein D (HNRNPD; also known as AUF1). In our previous research, postpartum administration of sodium salicylate (SS) increased whole-lactation milk yield in multiparous cows but tended to reduce milk yield in primiparous cows. Because rapid mammary tissue development likely occurs in cows approaching first lactation, we hypothesized that SS inhibited the activation of HIF-1α and decreased transcription of downstream targets. MAC-T cells were treated with SS (100 μ*M*) or control medium before incubation under either hypoxic (1% O_2_) or normoxic conditions for 12 h. Additionally, cells were transfected with either *HIF1A* small interfering RNA (siRNA) or a scrambled siRNA negative control 48 h before hypoxia treatments. *HIF1A, GLUT1, VEGFA*, and *HNRNPD* were quantified relative to the internal control gene *NENF*. Transcript abundance was assessed using a linear mixed model with the fixed effects of SS, hypoxia, siRNA, and all 2- and 3-way interaction terms and the random effect of plate nested within hypoxia. Treatment with SS interacted with hypoxia for *GLUT1*, as SS reduced *GLUT1* when MAC-T cells were cultured in normoxic conditions; however, no effect of SS was found in hypoxia-treated cells. Regardless of oxygen status, SS reduced *HNRNPD* and tended to decrease *VEGFA* mRNA relative to untreated cells. Hypoxia increased *GLUT1*, yet no effect was observed on *VEGFA* or *HNRNPD*. Small interfering RNA knocked down *HIF1A*, but no effect was found on *GLUT1, VEGFA*, or *HNRNPD*. In conclusion, SS reduced transcript abundance of genes involved with mammary gland development but generally did not interact with oxygen status.

## Highlights

• Sodium salicylate decreased abundance of transcripts involved in mammary development.
• Knockdown of HIF-1α did not prevent hypoxia-induced glucose transporter 1 expression.
• Few interactions between hypoxia and sodium salicylate were observed.

## Graphical Abstract

**Figure:**
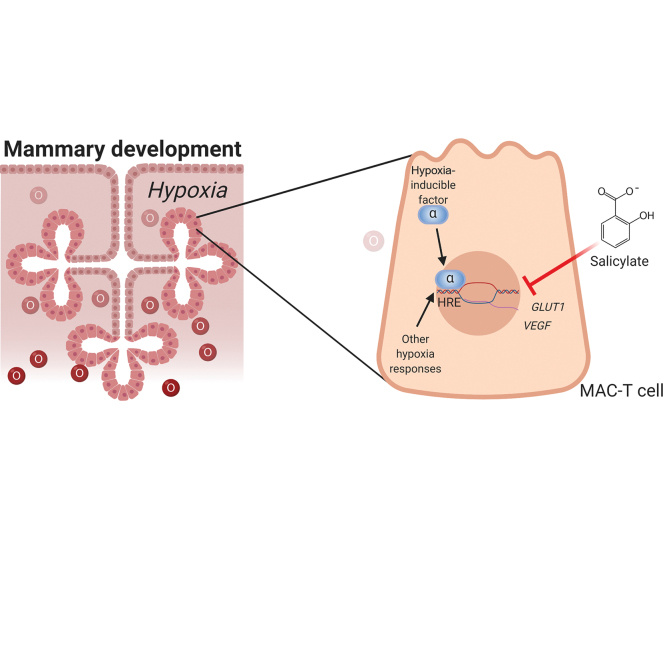
**Summary:** It is likely that transient hypoxia occurs with rapid development of the mammary gland during lactogenesis. Overlapping transcriptional responses to inflammatory stimuli and hypoxia point to potential interactions in regulation of tissue development. In the MAC-T mammary epithelial cell line, hypoxia induced expression of glucose transporter 1 (*GLUT1*), whereas the anti-inflammatory drug sodium salicylate decreased mRNA abundance of *GLUT1* and heterogeneous nuclear ribonucleoprotein D and tended to suppress expression of the paracrine vascular growth factor vascular endothelial growth factor A. Although interactions of hypoxia and salicylate were only detected for *GLUT1* abundance, salicylate treatment generally opposed previously reported transcriptional responses to hypoxia.

During late pregnancy, the mammary epithelium must rapidly expand to support lactation. Endocrine control on bovine mammary gland development has been extensively studied (Tucker, 2000); however, little is known about the effect of oxygen availability. Hypoxia is an oxygen deficiency commonly found in growing tissues and is speculated to occur in the mammary gland (Shao and Zhao, 2014). An increase in oxygen consumption was noted in the mammary gland of goats during late pregnancy and into lactation (Reynolds, 1967; Davis et al., 1979). During lactogenesis, oxygen demands of growing mammary tissue may outpace vascular growth and blood supply, potentially causing a transient local hypoxic environment. Low oxygen concentrations can activate hypoxia-inducible factor-1 (**HIF-1**). This transcription factor has 2 subunits, HIF-1α and HIF-1β, with the former being stabilized during hypoxic conditions and its expression highly regulated and the latter being constitutively expressed (Semenza, 2003). Activation of HIF-1 increases transcription of genes involved in angiogenesis, glucose transport, and cell survival and proliferation (Semenza, 2003). In mouse studies, mammary HIF-1α demonstrated an essential role in mammary gland development (Shao and Zhao, 2014), as knockout resulted in smaller alveoli and impaired epithelial cell differentiation, which consequently resulted in a dramatic reduction in milk synthesis (Seagroves et al., 2003).

The effect of hypoxia on milk synthesis may be partly explained by its effect on glucose transporters and angiogenesis. Indeed, hypoxia increased mRNA abundance of glucose transporter 1 (*GLUT1*) in mouse mammary epithelial cells (Seagroves et al., 2003). Likewise, MAC-T cells cultured under hypoxic conditions had a greater mRNA abundance of *GLUT1* compared with cells treated with normoxia (Shao et al., 2014). Moreover, *GLUT1* transcript abundance is greater during early to peak lactation compared with either nonlactation or late lactation in multiparous cows (Komatsu et al., 2005; Finucane et al., 2008; Mattmiller et al., 2011). Lactose synthesis, which requires glucose uptake, dictates milk volume (Zhao, 2014), and thus a hypoxia-induced increase in GLUT1 may be a mechanism to augment milk synthesis. Hypoxia also plays a critical role in angiogenesis by increasing transcription of vascular endothelial growth factor (**VEGF**), a paracrine function to increase delivery of oxygen and nutrients to growing tissues (Forsythe et al., 1996). Thus, hypoxia-induced effects, along with shifts in hormone levels around the time of parturition, are likely important driving factors for mammary gland development.

One potential mechanism behind the downstream transcriptional effects of hypoxia-associated genes is the recruitment of heterogeneous nuclear ribonucleoprotein D (**HNRNPD**); HNRNPD destabilizes mRNA of DNA methyltransferase 1 (**DNMT1**), an enzyme that catalyzes the addition of methyl groups to DNA. Destabilization of *DNMT1* mRNA causes global DNA hypomethylation (Torrisani et al., 2007; Hsiao et al., 2015), which effectively increases expression of the aforementioned hypoxia-associated genes (Wu et al., 2019). Moreover, in a cancer cell line, HNRNPD positively regulated VEGF and HIF-1α by directly binding and stabilizing their mRNA (Al-Khalaf and Aboussekhra, 2019). Heterogeneous nuclear ribonucleoprotein D may be a key regulator of genes associated with hypoxia and mammary gland development.

Although much is known about hypoxia-induced effects on glucose transporters and angiogenesis, an emerging body of research suggests that hypoxia itself is an inflammatory stimulus. Nuclear factor kappa-light-chain-enhancer of activated B cells (**NF-κB**) is a transcription factor that can be activated by hypoxia (Cummins and Taylor, 2005; Cummins et al., 2006; BelAiba et al., 2007; Rius et al., 2008), and *HIF1A* expression is regulated by NF-κB (van Uden et al., 2008). Nuclear factor-κB controls the expression of inflammatory mediators in addition to playing a critical role in cell proliferation, including mammary gland development (Cao and Karin, 2003). Elevated levels of inflammatory mediators are commonly observed in periparturient dairy cows (Bradford et al., 2015). However, excessive inflammation in early lactation has negative long-term effects on milk production (Bertoni et al., 2008). Administration of sodium salicylate (**SS**), a nonsteroidal anti-inflammatory drug (**NSAID**) that inhibits NF-κB activation (Kopp and Ghosh, 1994), for 7 d following parturition increased whole-lactation milk yield for cows in third or greater lactation but tended to decrease milk yield for first-parity cows (Farney et al., 2013b). During the beginning of a cow's first lactation, hypoxia-induced NF-κB activation is likely an important mechanism to promote mammary gland development. Thus, NSAID administration during a time of intense mammary gland development may explain the parity interaction noted in our previous study. Moreover, we recently found that SS administration increased mammary global DNA methylation (Ylioja et al., 2018), another hint that SS could alter hypoxia-induced effects. Therefore, the objective of the present study was to investigate the effect of SS on hypoxia-induced responses in MAC-T cells. We hypothesized that SS would inhibit the activation of HIF-1α, resulting in decreased transcription of downstream targets responsible for glucose transport (*GLUT1*) and angiogenesis (*VEGFA*) due to a reduction in mRNA abundance of *HNRNPD*, a gene involved in altering global DNA methylation patterns.

Immortalized bovine mammary epithelial (MAC-T) cells were kindly donated by Wendi Cohick from Rutgers University (Brunswick, NJ). Dulbecco's modified Eagle's medium (1×; ref no. 11965-092, Life Technologies) containing 10% fetal bovine serum (cat. no. A3160401, Thermo Fisher Scientific), 100 U/mL penicillin streptomycin (cat. no. 15070, Life Technologies), and 5 µg/mL insulin (cat. no. I9278; Sigma Aldrich) was used for growing cells and in all experiments. Cells were cultured at 37°C with 5% atmospheric CO_2_ in humidified incubators. Cell were passed at 80% confluency. Cells were first washed with Ca- and Mg-free PBS, and then TrypLE solution (cat. no. 12604013, Thermo Fisher Scientific) was added and cells were incubated for 2 to 5 min until cells were completely detached. Detached cells were resuspended in fresh medium, quantified with a Neubauer chamber, and plated at the desired cell density.

For experiments, MAC-T cells (10^6^ cells/well) were seeded into 12-well cell culture plates. Before treatments were applied, all cells were cultured for 24 h to reach at least 80% confluence. Afterward, cells were transfected with either *HIF1A* small interfering RNA (**siRNA**) or a scrambled siRNA negative control 48 h before treatment application. Cell were transfected in serum- and antibiotic-free Opti-MEM medium (cat. no. 11058021, Thermo Fisher Scientific) with a final concentration of 100 n*M* scrambled negative control (**NEG**) siRNA (Mission siRNA Universal Negative Control #1, cat. no. SIC001, Sigma Aldrich), *HIF1A* siRNA-1, or *HIF1A* siRNA-2 (Sigma-Aldrich) using Mirus TransIT-X2 Transfection Reagent (cat. no. MIR6000, MirusBio) according to the manufacturer's recommendations. Additionally, some wells underwent this process but without any siRNA duplex (**CON**). The siRNA duplex sequences were GGAUGAUGACUUCCAGUUAdTdT, UAACUGGAAGUCAUCAUCCdTdT for *HIF1A* siRNA-1 and CUGAUUUAGACUUGGAGAUdTdT, AUCUCCAAGUCUAAAUCAGdTdT for *HIF1A* siRNA-2.

After 48 h, cells were washed with PBS before fresh culture medium containing SS (100 μ*M*; cat. no. S3007, Sigma-Aldrich) or not was added just before incubation. This dose was based on the approximate mean plasma concentration of salicylate in postpartum cows treated with oral SS (unpublished data from Montgomery et al., 2019). A gas-tight modular incubator chamber (MIC-101, Billups-Rothenberg) was flushed for 3 min with 5% CO_2_ balanced with 95% nitrogen, resulting in an oxygen concentration of approximately 1%. Treated cells were immediately placed in this chamber to induce hypoxia (1% O_2_) or were placed outside the chamber in normoxic conditions in the incubator (5% CO_2_), as in previous studies (Joshi et al., 2011; Portou et al., 2020). Cells were then incubated under either hypoxic or normoxic conditions for 12 h (n = 6–7 wells per treatment combination).

After the 12-h incubation, the medium was removed and cells were washed with cold PBS. Cells were harvested with 0.5 mL of Trizol containing β-mercaptoethanol (1.14 μL/0.5 mL of Trizol), removed using a cell scraper, and stored at −20°C until RNA was harvested. The RNA was extracted (RNeasy Lipid Tissue Mini Kit, Qiagen) according to manufacturer specifications. Concentration and purity of RNA were assessed using spectrophotometry (Take3 Micro-Volume plate, Biotek). Total RNA (mean: 45 ng) was used to synthesize cDNA in a 20-µL reaction using a High-Capacity cDNA Reverse Transcription Kit (Applied Biosystems). Quantitative real-time PCR was performed in duplicate with 1 µL of the cDNA product in the presence of 200 nmol/L gene-specific forward and reverse primers using SYBR Green fluorescent detection (7500 Fast Real-Time PCR System, Applied Biosystems). Primers were designed using the Primer-BLAST tool on the National Center for Biotechnology Information website (https://www.ncbi.nlm.nih.gov/tools/primer-blast/; Table 1). Efficiencies of PCR were determined using a 5-point dilution curve and ranged between 92 and 110% for all gene targets (Table 1), and transcript abundance was quantified using the relative expression ratio incorporating efficiency from Pfaffl (2001). Numerous housekeeping genes were checked for stability across treatments. Potential internal control gene transcripts *RPS9, RPS15, UXT*, and neudesin neurotrophic factor (*NENF*) were evaluated for stability across treatments; *NENF* was selected as the most appropriate internal control because it was readily detectible in all samples, was not altered by treatment (*P* = 0.67), and showed greater stability across samples than the geometric mean of 3 reference genes (Pfaffl et al., 2004). Specificity of primer amplification was verified by melt curve analysis following PCR.

**Table 1:** Primers used for quantitative PCR analysis

Gene	Accession number	Primer^1^	Region amplified (bp)	Reaction efficiency (%)
*NENF*	NM_001076419.1	F: TCAAGGGGGTGGTGTTCGAT	206–255	90
		R: TCGTCCATAAAACTCCTTTCCAG		
*HIF1A*	XM_024997269.1	F. GGATGATGACTTCCAGTTA	2,837–2,855	110
		R: TAACTGGAAGTCATCATCCA		
*GLUT1*	NM_174602.2	F: ACTCCATCATGGGCAACCAG	700–837	92
		R: GGTTCTCCTCGTTGCGGTTA		
*VEGFA*	NM_001316956.1	F: AGAGATGAGCTTCCTACAGCA	1,398–1,540	106
		R: GAGCGCTCCAGGATTTATACC		
*HNRNPD*	XM_005208170.3	F: AACGAGGAGGATGAAGGGAAAA	516–621	105
		R: TGCAGTCTACGACTTCACCA		
*RPS9*	XM_024978366.1	F: CAGCTCTCCTTCTCGCACAG	90–219	93
		R: TACTCGCCGATCAGCTTCAG		
*RPS15*	NM_001024541.2	F: ATGGTTGGCGTCTACAACGG	297–353	88
		R: CATCTCAGGCTTGATTTCCACC		
*UXT*	NM_001037471.2	F: ATTGAGCGACTCCAGGAAGC	196–281	92
		R: GGGACCACTGTGTCAACGAA		

1 F = forward; R = reverse.

MAC-T cells (10^6^ cells/well) were seeded into a 96-well cell culture plate and were treated with SS (100 μ*M*) or control medium just before incubation under either hypoxic (1% O_2_) or normoxic conditions for 12 h (n = 6 per treatment combination). AlamarBlue was incubated with MAC-T cells for 4 h, and the conversion of resazurin to resorufin was used as a proxy for cell viability (cat. no. DAL1025, Invitrogen). Absorbance was determined at 570 nm using a plate reader (Synergy HTX, BioTek Instruments Inc.) and Gen5 software (BioTek Instruments Inc.).

For cell viability, a linear mixed model (PROC GLIMMIX of SAS 9.4, SAS Institute Inc.) was used with hypoxia, SS, and the interaction as fixed effects and the random effect of cell culture plate nested within hypoxia treatment. Transcript abundance of 4 target genes (*HIF1A, GLUT1, VEGFA*, and *HNRNPD*) was analyzed using a linear mixed model (PROC GLIMMIX) with the fixed effects of SS, hypoxia, siRNA, and all 2- and 3-way interaction terms and the random effect of cell culture plate nested within hypoxia treatment. To meet the assumption of normality (PROC UNIVARIATE of SAS 9.4), all mRNA abundance data required natural logarithmic transformation. Least squares means and standard errors were back-transformed according to Jørgensen and Pedersen (1998). An outlier was defined if the observation had a studentized residual greater than 3 in absolute value, and therefore was removed from the analysis. When conducting multiple comparisons, treatment means were separated using Tukey's honestly significant difference test. Significance was declared at *P* ≤ 0.05 and tendencies were declared at 0.05 < *P* < 0.10.

For cell viability, the effect of SS depended on the oxygen concentration (Figure 1; SS × hypoxia, *P* < 0.01). Further investigation into this interaction yielded marginal effects of SS on cell viability, where SS increased cell viability under hypoxic conditions (*P* = 0.05) and decreased cell viability when cells were cultured in normoxia conditions (*P* < 0.01). In untreated cells, hypoxia marginally decreased cell viability compared with normoxia cells (*P* = 0.02); however, this cytotoxic effect was not found within the SS-treated cells (*P* = 0.97).

**Figure 1:**
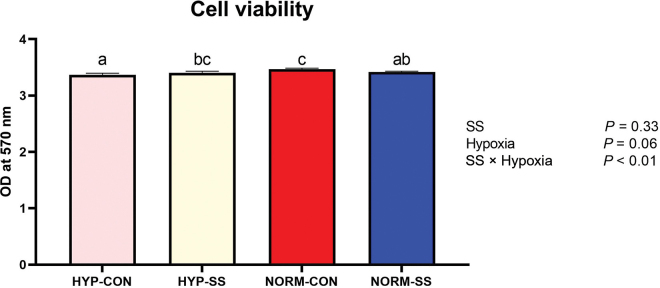
Marginal effects of sodium salicylate (SS) and hypoxia (HYP) on MAC-T cell viability. Sodium salicylate slightly increased cell viability, as measured by resazurin metabolism, under hypoxic conditions (*P* = 0.05) and decreased cell viability when cells were cultured in normoxia (NORM) conditions (*P* < 0.01). In untreated cells, HYP marginally decreased cell viability compared with NORM (*P* = 0.02). Different letters (a–c) indicate significant differences (*P* ≤ 0.05). CON = control; OD = optical density.

Neither SS nor hypoxia had clear effects on *HIF1A* (Figure 2A; *P* = 0.14 and *P* = 0.15, respectively). Statistical contrasts were conducted to evaluate the efficacy of 2 *HIF1A* siRNA. Both siRNA-1 and siRNA-2 successfully knocked down *HIF1A* (CON and NEG vs. siRNA-1 and -2; *P* < 0.01), and both siRNA had similar efficacies (siRNA-1 vs. siRNA-2, *P* = 0.19). To assess downstream hypoxia-induced transcriptional effects, relative mRNA abundance of *GLUT1, VEGFA*, and *HNRNPD* (Figure 2B–D) was quantified. Sodium salicylate interacted with hypoxia to influence transcript abundance of *GLUT1* (SS × hypoxia, *P* = 0.05; Figure 2B). Contrasts were conducted to evaluate the effect of SS within oxygen availability as well as the effect of hypoxia within NSAID treatment. Salicylate treatment reduced *GLUT1* when MAC-T cells were cultured in normoxia conditions (normoxia SS vs. normoxia CON, *P* < 0.01); however, no effect of SS was found when MAC-T cells were cultured in hypoxia conditions (hypoxia SS vs. hypoxia CON, *P* = 0.39). As expected, hypoxia increased *GLUT1* (hypoxia SS vs. normoxia SS, *P* < 0.01; hypoxia CON vs. normoxia CON, *P* = 0.01); however, no effect of *HIF1A* siRNA knockdown was found (*P* = 0.98; Figure 2B). No effect of either hypoxia or *HIF1A* knockdown by siRNA was identified for *VEGFA* (*P* ≥ 0.52; Figure 2C) or *HNRNPD* (*P* ≥ 0.52; Figure 2D). However, SS tended to reduce *VEGFA* (*P* = 0.06; Figure 2C) and decreased *HNRNPD* (*P* = 0.03; Figure 2D) transcript abundance compared with untreated cells.

**Figure 2:**
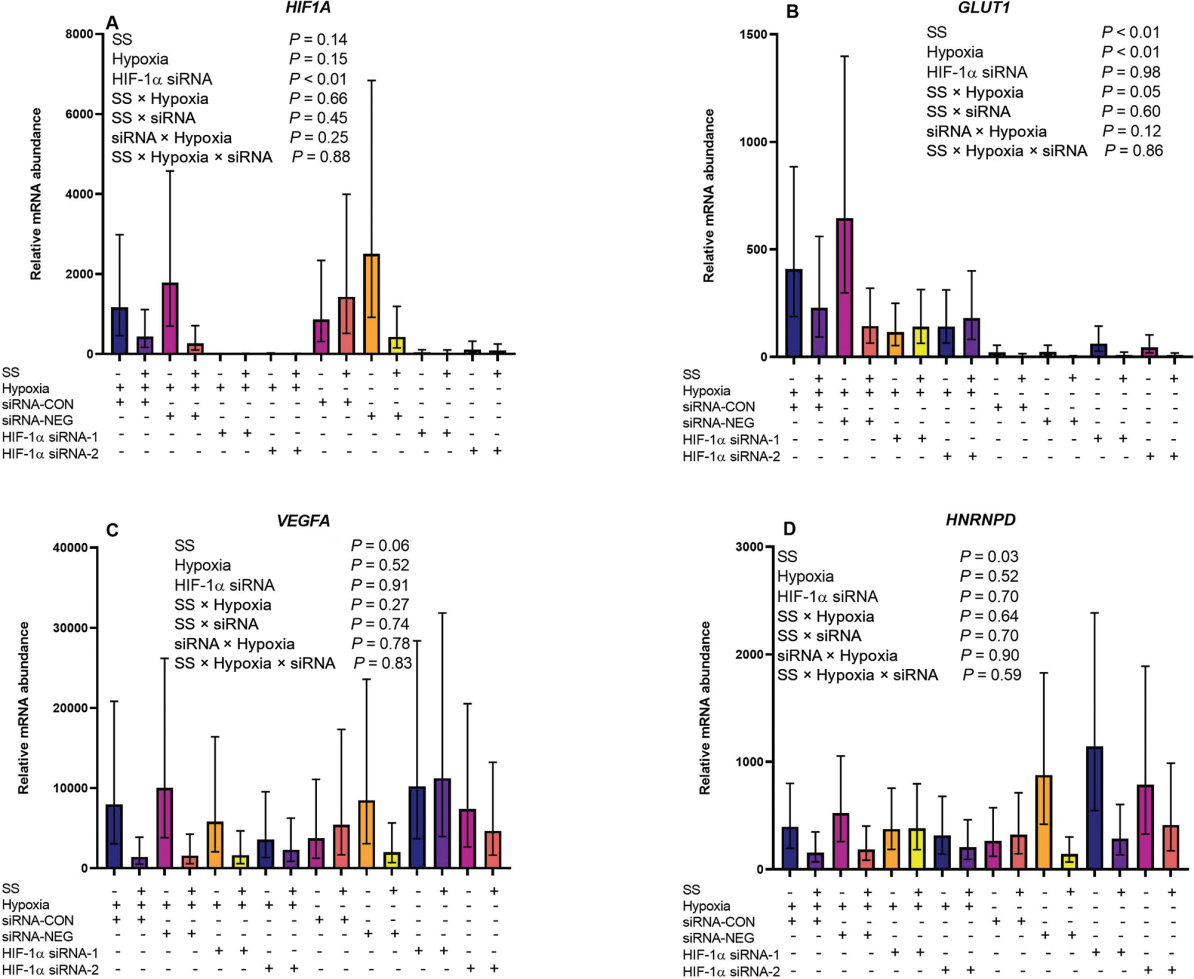
Sodium salicylate (SS) had marginal effects on the transcription factor hypoxia-inducible factor-1 (HIF-1) in MAC-T cells but significantly reduced transcript abundance of downstream targets. Back-transformed LSM (±SE) are provided for *HIF1A* (A), *GLUT1* (B), *VEGFA* (C), and *HNRNPD* (D) transcript abundance relative to *NENF*. (A) Small interfering RNA (siRNA)-1 and siRNA-2 successfully knocked down *HIF1A* (CON and NEG vs. siRNA-1 and siRNA-2, *P* < 0.01), and both siRNA had similar efficacies (siRNA-1 vs. siRNA-2, *P* = 0.19). CON = no siRNA control; NEG = scrambled siRNA negative control.

We hypothesized that SS would reduce mRNA abundance of downstream targets associated with hypoxia through inactivation of HIF-1α. Although salicylate failed to alter *HIF1A* mRNA, NSAID effects were more apparent in downstream targets as SS decreased *GLUT1* in normoxic cells and tended to decrease *VEGFA* mRNA regardless of oxygen status. Hypoxia induces inflammation via NF-κB, which in turn activates HIF-1 (Hellwig-Bürgel et al., 1999; van Uden et al., 2008). A reduction in HIF-1α and its downstream targets is likely due to NF-κB sequestration in the cytosol by SS. Numerous studies have demonstrated a protective role for HIF-1 in the gut epithelium (Karhausen et al., 2004; Rosenberger et al., 2009; Clambey et al., 2012), likely to restore tissue homeostasis. These data lead us to speculate that HIF-1α exerts an anti-inflammatory effect in mammary alveolar cells, and thus the reduction in HIF-1α-related targets by SS treatment likely resulted from a reduced proinflammatory status.

In contrast to our hypothesis, hypoxia did not increase *HIF1A* mRNA abundance in MAC-T cells, although it is important to note that activation of HIF-1 activity in vivo is not necessarily dependent on enhanced *HIF1A* transcription. We also tested responses to 2 anti-*HIF1A* siRNA duplexes. Although both siRNA successfully reduced *HIF1A* mRNA abundance, no downstream effects were found on *GLUT1, VEGFA*, or *HNRNPD*. The lack of obvious downstream effects may be due to redundant transcription regulation of these genes by numerous transcription factors. For instance, in addition to HIF-1, VEGF (Jośko and Mazurek, 2004) and GLUT1 (Kao and Fong, 2008) are controlled by the transcription factor Sp1.

Salicylate in the present study reduced *GLUT1* in normoxia, but no effect of SS was noted in cells cultured under hypoxic conditions. A combination of in vivo and in vitro evidence suggests that GLUT1 is the dominant glucose transporter in bovine mammary epithelial cells (Zhao and Keating, 2007), so suppression of GLUT1 transcription in early-lactation cows would likely constrain glucose availability to support milk lactose synthesis and, to a lesser extent, synthesis of other milk components. However, in our previous studies, oral SS administration reduced blood glucose concentration in multiparous cows due to impaired gluconeogenesis (Montgomery et al., 2019) but had no effect on lactose or milk yield during NSAID treatment (Farney et al., 2013a), which does not align with a central role of mammary *GLUT1* in SS responses in vivo.

One potential mechanism behind the downstream transcriptional responses to hypoxia is epigenetics, which is the alteration of gene expression due to chemical modification of DNA or histones. One epigenetic modification is DNA methylation, which controls gene expression by hindering transcription factor binding and consequently repressing transcription (Riggs et al., 1996; Jaenisch and Bird, 2003). As previously mentioned, hypoxia triggers HNRNPD activation, which destabilizes mRNA of the enzyme DNMT1. This enzyme catalyzes the addition of methyl groups to CpG structures in DNA. Thus, a reduction of DNMT1 results in selective DNA hypomethylation, leading to increased expression of hypoxia-associated transcripts (Wu et al., 2019). Salicylate administration in a previous study increased mammary global DNA methylation in multiparous cows (Ylioja et al., 2018), possibly driven by a reduction in HNRNPD. Although we are unsure of how SS reduces HNRNPD transcript abundance, it may be due to its anti-inflammatory and antioxidant properties (Franco et al., 2008). The unraveling of NSAID epigenetic effects in the mammary gland and on dairy cow productivity could be a fruitful area of research (Singh et al., 2010).

We used an in vitro approach here to allow us to narrowly investigate direct interactions between hypoxia and SS without complicating endocrine and other regulatory effects in vivo. Furthermore, we chose to use the MAC-T cell line as a well-characterized model for mammary epithelial cells. However, this cell line does not respond exactly as primary bovine mammary epithelial cells do (Zavizion et al., 1995; Jedrzejczak and Szatkowska, 2014), and possible discrepancies between our findings and the in vivo scenario should be considered.

In conclusion, salicylate reduced mRNA abundance of genes involved with mammary tissue growth and development in MAC-T cells. Future in vivo studies should examine the interactions of NSAID therapy and parity on HIF-1α abundance and its downstream targets in the mammary gland.
